# Apramycin treatment affects selection and spread of a multidrug-resistant *Escherichia coli* strain able to colonize the human gut in the intestinal microbiota of pigs

**DOI:** 10.1186/s13567-015-0291-z

**Published:** 2016-01-07

**Authors:** Ana Herrero-Fresno, Camilla Zachariasen, Monica Hegstad Hansen, Alexander Nielsen, Rene S. Hendriksen, Søren Saxmose Nielsen, John Elmerdahl Olsen

**Affiliations:** Department of Veterinary Disease Biology, Faculty of Health and Medical Sciences, University of Copenhagen, Frederiksberg, Denmark; WHO Collaborating Centre for Antimicrobial Resistance in Food-borne Pathogens and EU Reference Laboratory for Antimicrobial Resistance, National Food Institute, Technical University of Denmark, Kongens Lyngby, Denmark; Department of Large Animal Sciences, Faculty of Health and Medical Sciences, University of Copenhagen, Frederiksberg, Denmark

## Abstract

The effect of apramycin treatment on transfer and selection of an *Escherichia coli* strain (*E*. *coli* 912) in the intestine of pigs was analyzed through an in vivo experiment. The strain was sequenced and assigned to the sequence type ST101 and serotype O11. It carried resistance genes to apramycin/gentamicin, sulphonamide, tetracycline, hygromycin B, β-lactams and streptomycin [*aac*(*3*)-*IV*, *sul2*, *tet*(*X*), *aph*(*4*), *bla*_TEM-1_ and *strA/B*], with all but *tet*(*X*) located on the same conjugative plasmid. Nineteen pigs were randomly allocated into two inoculation groups, one treated with apramycin (pen 2) and one non-treated (pen 3), along with a non-inoculated control group (pen 1). Two pigs of pen 2 and 3 were inoculated intragastrically with a rifampicin resistant variant of the strain. Apramycin treatment in pen 2 was initiated immediately after inoculation. Strain colonization was assessed in the feces from all pigs. *E*. *coli* 912 was shown to spread to non-inoculated pigs in both groups. The selective effect did not persist beyond 3 days post-treatment, and the strain was not detected from this time point in pen 2. We demonstrated that *E*. *coli* 912 was able to spread between pigs in the same pen irrespective of treatment, and apramycin treatment resulted in significantly higher counts compared to the non-treated group. This represents the first demonstration of how antimicrobial treatment affects spread of resistant bacteria in pig production. The use of apramycin may lead to enhanced spread of gentamicin-resistant *E*. *coli*. Since gentamicin is a first-choice drug for human bacteremia, this is of concern.

**Electronic supplementary material:**

The online version of this article (doi:10.1186/s13567-015-0291-z) contains supplementary material, which is available to authorized users.

## Introduction

During the last 50 years, antibiotics have been used to treat infections in both human and veterinary medicine. In this period, use of antimicrobials has caused selective evolutionary pressures, resulting in the emergence of antimicrobial resistant bacteria [1], which in turn have caused treatment failure and increased morbidity [2]. The natural gut microbiota, consisting of commensal bacteria, represents an important reservoir for the development of antimicrobial resistance (resistome), and continuous antibiotic use could lead to the emergence of clinically problematic strains [3].

Multi-drug resistant *E*. *coli* isolates from humans and pigs have been reported worldwide [4–7]. These multi-drug resistant isolates harbor antimicrobial resistance (AMR) genes either on the chromosome or on mobile genetic elements, such as plasmids [8]. The presence of AMR genes on plasmids, and their subsequent horizontal transfer via conjugation, can result in their rapid spread among the susceptible bacterial populations [9]. One of the main concerns is the potential transfer of these resistant determinants to pathogenic bacteria which prolongs infections and decreases treatment options as a consequence.

Use of antibiotics in livestock is considered one of the main reasons leading to development of AMR, and such resistant bacteria can be potentially transmitted to the food chain [2]. The role of pigs in AMR transmission to the food chain must be better understood in order to prevent dissemination of multidrug-resistant strains from pigs to humans. In a previous study a multi-drug resistant strain of *E*. *coli* isolated from a healthy pig (*E*. *coli* 912) [10] was proven to be able to colonize the gut of human volunteers for 35 days [11]. The strain harbored the resistance gene *aac*(*3*)-*IV* conferring resistance to gentamicin/apramycin [11].

Apramycin, which is only approved for animal use, was introduced into veterinary therapy in the early 1980s in several European countries [12]. Several apramycin products were authorized for oral use in production animals in 1998 [13]. The gene *aac*(*3*)-*IV* is the only identified gene causing enzymatic cross-resistance between gentamicin and apramycin [12]. Apramycin resistance associated with the *aac*(*3*)-*IV* gene was initially reported in 1981 in France, and the gene was only found in the animal reservoir [14]. Over the next years *aac*(*3*)-*IV* disseminated rapidly in the animal reservoir in France, Belgium and Great Britain [14]. In 1986, the gene was first detected in *Enterobacteriaceae* isolated from human patients [15].

Nowadays, apramycin is widely used in farm animals, and resistant *E*. *coli* are commonly isolated from diseased pigs [10]. *E*. *coli* from pigs may be an important reservoir for transfer of apramycin/gentamicin resistance genes or bacteria to humans. Furthermore, resistance to apramycin and other aminoglycosides is usually transmissible, encoded on conjugative R-plasmids, and often linked to resistance to other antimicrobials [12].

In Denmark, detailed information on aminoglycoside use in food-producing animals is registered in the Danish veterinary drug-monitoring programme, VetStat [16]. This database contains information on consumption of all prescription drugs purchased by animal owners or used by veterinarians at farm level, including information on animal species, age group, disease group and farm identity. Importantly, gentamicin is a first-choice drug (in combination with β-lactams) for severe human infections (e.g. sepsis and endocarditis) in Danish Hospitals [17]. Therefore, spread of gentamicin-resistant *E*. *coli* strains to humans is of great concern.

Several studies have evaluated the impacts of antimicrobial treatment on selection for resistance [18–23], however, only a few reports have considered the impact of treatment on the spread of resistant microorganisms among animals [24, 25]. Here, we quantify for the first time the effect of treatment on the transmission of resistant strains among pigs in vivo. In this study, the effect of apramycin treatment on the selection of the *E*. *coli* 912 inoculated into nursery pigs was assessed. Derived results provided information of the consequence of antibiotic treatment in the development and spread of resistant bacteria between pigs in production-like conditions (regular farm conditions), where pigs are housed closely together.

## Materials and methods

### Animals, housing conditions, and ethical issues

Three to 4 weeks nursery cross-bred sex-mixed pigs with weights ranging from 6 to 9 kg were purchased from a specific-pathogen-free farm in Denmark. The animals were housed in a level 1 isolation unit (individual disinfected pens in a same room of the building) at the Faculty of Health and Medical Sciences, University of Copenhagen and weighed at least once a week. All procedures regarding the animal experiments were carried out in agreement with the Animals Scientific Act and performed under the license and approval of the Danish National Animal Experiment Inspectorate (license no. 2009/561-1675). Occurrence of any clinical sign, such as changes in behavior and decrease in food and water intake, was surveyed twice a day. At the end of the experiment (3 weeks of duration), the animals were euthanized by captive bolt pistol penetration followed by exsanguination.

### Bacterial strain

The bacterial strain *E*. *coli* 912 used to inoculate the pigs was previously proven to be resistant to gentamicin/apramycin and sulphonamide by determination of minimum inhibitory concentration [10, 11]. It was isolated from the feces of a healthy pig [10, 11]. The strain harbored the genes *aac*(*3*)-*IV* and *sul2* on a conjugative plasmid (not shown) [11]. A rifampicin (RIF) resistant mutant was obtained by serial plating on nutrient agar with rifampicin. The RIF-resistance (MIC ≥ 250 μg/mL) was used as a marker to distinguish the inoculated strain from gentamicin/sulphonamide-resistant coliforms that could preexist in the intestinal microbiota or that emerged during the experiment as a result of horizontal gene transfer. Growth of both wild-type and the isogenic RIF-resistant mutant strain was analysed and compared. The isolates were grown at 37 °C, 200 rpm for 16 h in Luria–Bertani broth before sub-culturing into fresh media at a 40 fold dilution and further grown with assessments every 15 min for 18 h using Bioscreen C. Growth curves were obtained (Additional file 1).

### Whole genome sequencing, analysis of genome sequence for virulence, resistance, serotype, plasmid associated genes, and multilocus sequence typing (MLST)

Genomic DNA was extracted from the isolate *E*. *coli* 912 using the Invitrogen Easy-DNATM Kit (Invitrogen) and DNA concentrations were determined using the Qubit dsDNA BR assay kit (Invitrogen). The genomic DNA was prepared for Illumina pair-end sequencing using the Illumina (Illumina) NexteraXT^®^ Guide 150319425031942 following the protocol revision C [26]. A sample of the pooled NexteraXT Libraries was loaded onto an Illumina MiSeq reagent cartridge using MiSeq Reagent Kit v2 and 500 cycles with a Standard Flow Cell. The libraries were sequenced using an Illumina platform and MiSeq Control Software 2.3.0.3. The isolate was pair-end sequenced.

The raw reads were assembled using the Assemble pipeline (version 1.0) available from the Center for Genomic Epidemiology (CGE) [27] which is based on the Velvet algorithms for de novo short reads assembly. The assembled genome was submitted and annotated at NCBI [28] under accession number JWJM00000000.

The assembled sequences were analyzed to confirm the species and *E*. *coli* serotype using the CGE pipelines; K-merFinder (version 2) [29] and SeroTypeFinder (version 1.1). Following the confirmation, the MLST sequence type (ST) for *E*. *coli*, plasmid replicons, and acquired antimicrobial resistance genes, and virulence genes were identified with a selected threshold equal to 98% identity using the pipelines; MLST (version 1.8) [30], PlasmidFinder (version 1.3) [31], ResFinder (version 2.1) [32], and VirulenceFinder (version 1.4) [33] also available from CGE.

### Plasmid analysis and Southern blot

Plasmid DNA from *E*. *coli* 912 was isolated with the MidiPrep plasmid kit (Invitrogen) following the protocol suggested by the manufacturer. DIG-labelled DNA molecular weight marker II (Roche) was used as molecular size standard and control in Southern blot hybridization. The obtained plasmid profile was subsequently hybridized with probes specific for the *sul2*, *aac*(*3*)-*IV*, *tet*(*X*), *bla*_TEM-*1*_ and *strA/B* genes by using the kit DIG wash and block buffer set (Roche) and manufacturers indications from the DIG application manual for filter hybridization. The probes were obtained from *E*. *coli* 912 by PCR amplification using the PCR DIG labelling mix (Roche). The sequences of the primers used for PCR are listed in Table 1.

**Table 1:** Primers used in this study.

Primers	Sequence (3′–5′)
aac(3)-IV-F	AGTTGACCCAGGGCTGTCGC
aac(3)-IV-R	GTGTGCTGCTGGTCCACAGC
bla_TEM_-F	TTTGCGGCATTTTGCCTTCCT
bla_TEM_-R	GTTCATCCATAGTTGCCTGAC
strA-F	TTG ATG TGG TGT CCC GCA ATG C
strA-R	CCA ATC GCA GAT AGA AGG CAA
sul-2-F	TTTCGGCATCGTCAACATAA
sul-2-R	GTGTGTGCGGATGAAGTCAG
tet(X)-F	TTAGCCTTACCAATGGGTGT
tet(X)-R	CAAATCTGCTGTTTCACTCG

### Challenge experimental setup

Pigs were divided into three groups housed in three well-separated pens avoiding any contact among pigs from different pens. Only airflow between pens was possible. The untreated control group (*n* = 3) was isolated in pen 1. The two inoculated groups, group 2 (pen 2) and group 3 (pen 3) included eight pigs each. After 1 week of acclimatization, two pigs from each of groups 2 and 3 were inoculated with 10^9^ CFU/mL of the *E*. *coli* 912 strain suspended in 10 mL of a saline suspension using an endogastric tube after sedation. The four inoculated pigs were isolated in an individual pen for 2 days in order to allow the bacterial colonization of the gut and then replaced in their original groups.

The antimicrobial drug, Apralan Vet 10% solution, was purchased as veterinary medical product. All pigs in group 2 were individually treated with 5 mg/kg of the antibiotic after the replacement of the two previously inoculated pigs, and the antimicrobial product was administered once a day for 3 days orally in nutri-drink ensuring that everything was taken up. The administration was performed according to the standard treatment recommended when administering the drug product in pigs.

### Collection and microbiological analysis of fecal samples

Fecal samples of about 5 g were collected from the rectum of all the pigs prior to inoculation of the strain (day −2), 1 day after inoculation (day −1), prior to antimicrobial treatment in pen 2 (day 0) and on days 2, 4, 6, 8, and 10 after day 0 (days corresponding last day of treatment-day 2- and 2, 4, 6 and 8 days after the end of the treatment). Except for day −1, where CFU counts were only performed for the four inoculated pigs, fecal samples were taken from all the 19 pigs and CFU counts were carried out. Serial tenfold dilutions were used to count the numbers of colony forming (CFU) coliforms on MacConkey agar (Merck), CFU of RIF-resistant coliforms on MacConkey agar supplemented with 50 μg/mL RIF, and CFU of the inoculated sulphonamide-gentamicin/apramycin (SUL-GEN/APRA) resistant strain on MacConkey agar supplemented with 50 μg/mL RIF, 150 μg/mL SUL and 25 μg/mL GEN. All counts were determined by the spot method [34]. Briefly, 30 μL of each dilution was inoculated as a spot in duplicate (on two plates containing every specific antibiotic or combination, and without antibiotic), followed by 24 h of incubation at 37 °C. This method allowed the detection of the coliforms and the quantification of the coliforms at greater than or equal to 500 CFU/g (2.7 log CFU/g) of feces.

On all days except -1, colonies were isolated from the selective plates containing RIF or RIF-SUL-GEN. All isolates were identified as *E*. *coli* by the indole, methyl red, Voges-Proskauer, and citrate tests. Isolates confirmed to be *E*. *coli* were tested for the presence of the *aac*(*3*)-*IV* gene by PCR (Table 1). In order to enable a comparison of the isolates obtained at different time points and the inoculated strain, all the RIF or RIF-SUL-GEN resistant isolates obtained from each animal of each group at all the sampling times were further characterized by pulsed-field gel electrophoresis (PFGE) with *Xba*I (Roche) digestion as previously described [35].

### Statistical analysis

The log CFU counts were compared by one-way ANOVA with pair-wise comparison of means at the different time points using Tukey’s multiple comparison test, while accounting for repeated measurements. A *P* value <0.05 was considered significant.

All occurrences in the individual pig on specific test days were further dichotomized (above or below detection level), which also enabled the estimation of incidence and recovery rates. For each of the time periods 0–2, 2–4, 4–6, 6–8 and 8–10 days post inoculation, the incidence rate (IR) was estimated using the formula [36]:

$$ IR = \frac{no.\;of\;cases}{{\left( {no.\;of\;pigs\;at\;risk\;at\;start\;of\;study\;period\; - \;0.5\; \times \;no.\;of\;pigs\;aquiring\;resistance\;in\;period} \right)\; \times \;time}} $$

The recovery rates (RR) were calculated similarly:

$$ RR = \frac{no.\;of\;recovered}{{\left( {no.\;of\;pigs\;at\;without\;resistance \;at\;start\;of\;study\; - \;0.5\; \times \;no.\;of\;pigs\;losing\;resistance\;in\;period} \right)\; \times \;time}} $$

## Results

### Traits of the strain *E*. *coli* 912

First the inoculated strain *E*. *coli* 912 was characterized in order to obtain information that could explain its high ability to colonize the gut of humans, as reported [11]. The strain was sequenced and found to contain additional resistance genes to the ones previously reported [11]. The additional genes encoded resistance to tetracycline [*tet*(*X*)], hygromycin B [*aph*(*4*)], β-lactams (*bla*_TEM-1_) and streptomycin (*strA/B*). This genotype complied with the phenotypic resistance results. The strain was shown to belong to MLST-type ST101 and serotype O11:H12. *E*. *coli* 912 also harbored the virulence genes *mchB*, *mchF*, *mcmA* (involved in microcin synthesis) *iroN* (iron uptake), *tsh* (hemoglobin binding protease), *cnf1* (toxin synthesis), *lpfA*, *prfB* (fimbriae synthesis) and *iss* (increased serum survival) (Table 2) and two plasmids belonging to the incompatibility groups *inc*F1 and *inc*FII. Since genome sequencing did not reveal information about the location of the resistance genes, plasmid purification followed by Southern blot hybridization was performed. Results showed that the genes *aph*(*4*), *bla*_*TEM*-*1*_*and strA/B* were plasmid located as it was previously found for *sul2* and *acc*(*3*)-*IV* [11]. Furthermore, all the five genes were harbored in the same resistance plasmid (not shown).

**Table 2:** Features of the strain. *E*. *coli* 912.

Strain used in this study	R-genes (phenotype)	V-genes (phenotype)	Plasmids	MLST-type	Serotype
*E*. *coli* 912	*bla*_*TEM*-*1*_ (β-lactams) *aac*(*3*)-*IV* (aminoglycoside) *aph4* (aminoglycoside) *strA/B* (aminoglycoside) *sul*-*2* (sulphonamide) *tetX* (tetracycline)	*mchB* (microcin) *mchC* (microcin) *mchF* (microcin *mcmA* (microcin) *iroN* (siderophore) *tsh* (serin protease autotransporter) *cnf1* (toxin) *lpfA* (fimbrae) *prfB* (fimbrae) *iss* (increased serum survival)	IncFII IncFIB	ST101	011:H12

A growth curve for each of the strains was obtained through in vitro studies and growth was not significantly different between the mutant and WT strains reaching the stationary phase after approximately 6–8 h post-inoculation (Additional file 1).

### The influence of apramycin treatment on spread and selection of the tested strain

**Figure 1:**
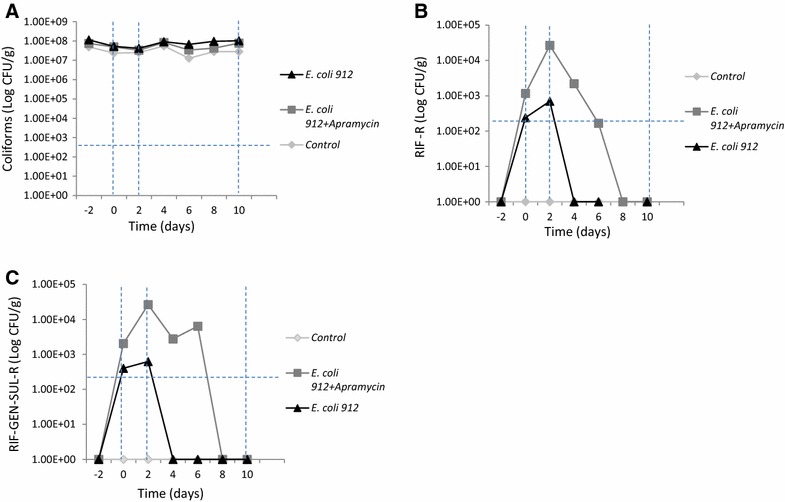
**Mean counts [log(x** **+** **1) CFU/g] of coliforms.** Counts of total coliforms (**A**), RIF-resistant coliforms (RIF-res) (**B**), and RIF-GEN-SUL-resistant coliforms (RIF-GEN-SUL-res) (**C**) in feces of pigs treated with apramycin, feces of untreated pigs and feces of pigs non-treated and non-inoculated (control). The vertical dashed lines indicate the treatment period (days 0–2) and the post treatment window (days 3–10). The horizontal dashed lines indicate the detection limit of the method used (500 CFU/g).

All experimental animals maintained good health status throughout the experiment, and their weights increased from 6–9 kg to 12–16 kg (average daily weight gain, 309 ± 0.052 g). The outline of the study is shown in Additional file 3 with the status of the individual pig at each time point. No significant differences in the average counts of total coliforms were observed between the treated group, the non-treated group and the control group (Figure 1A). Prior to inoculation, the feces of the 19 pigs did not contain neither RIF nor RIF-SUL-GEN resistant strains (strains growing after 18 h of growth at 37 °C). The counts of RIF and RIF-SUL-GEN resistant coliforms were significantly higher in the treated group (pen 2) than in the non-treated group (pen 3) from day 2 to day 6 after day 0 (start of treatment) (Figure 1B). The effect of treatment was evident until day 6 (4 days after the end of treatment which corresponded to day 2) and the highest peak was reached on the last day of treatment (2 days after day 0) (Figures 1B and C; Additional file 3). A higher number of pigs (up to seven out of eight-treated group vs. two out of eight-untreated group) tested positive for the strain when apramycin was administered (pen 2) (Additional file 3). Using plates with RIF-SUL-GEN for detection, the strain was observed in five and three pigs in the treated and the untreated group (Additional file 3B), respectively, while seven vs. four pigs were shown to excrete the strain when only RIF was used in the plate (Additional file 3A). RIF-SUL-GEN-positive strains were not recovered after day 6 (compared to day 0) in both groups, except that *E*. *coli* 912 was found in two pigs from pen 3 (untreated group) when only RIF was used for selection (Figure 1B; Additional file 3) on day 8 (with regards to day 0) but at very low numbers (<1 × 10^3^ cfu/mL). The presence of *E*. *coli* 912 was confirmed by PFGE. Thirty-six RIF and 36 RIF-SUL-GEN resistant isolates from days 0 (when the treatment was started and corresponding only to the inoculated pigs), 2 (last day of treatment), 4 (2 days after the end of treatment), 6 (4 days after the end of treatment) and 8 (6 days after the end of treatment) were identified as *E*. *coli*, and the presence of *aac*(*3*)-*IV* in all of them was confirmed by PCR. All isolates had the same *Xba*I-profile by PFGE, which corresponded to the profile of *E*. *coli* 912 (Additional file 2), thereby confirming that the counts on these selective agar plates were representative of the inoculated strain. None of the pigs in the unexposed groups showed RIF or RIF-SUL-GEN resistant bacteria.

**Figure 2:**
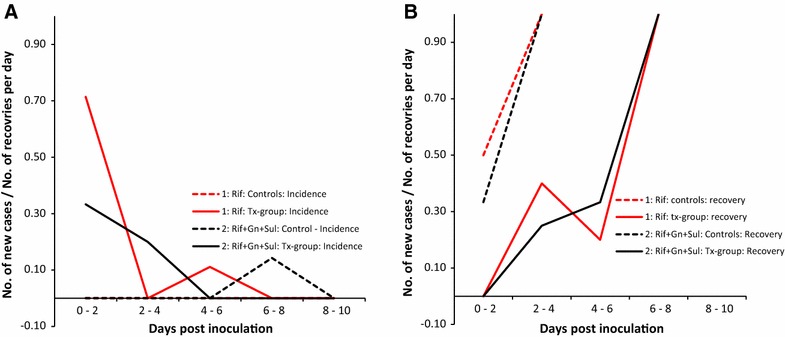
**Incidence and recovery rates.** Incidence rates (**A**) and recovery rates (**B**) for the different time periods, control groups and experiments.

The resulting incidence and recovery rates are displayed in Figures 2A and B, respectively. The specific rates are listed in Table 3. The incidence rate was high in the first time step for both RIF and RIF-GEN-SUL (Figure 2A), but 0 in both control groups. Recovery appeared almost identical in all groups, irrespective of the time points (Figure 2B). Statistical testing was not done because there was only one pen with each treatment and the separation in resistant and non-resistant counts was reasonably clear. Even though no significant difference was identified, the spread of *E*. *coli* 912 among pigs in the Apramycin treated group appeared higher than in the not treated group (Additional file 3).

**Table 3:** Incidence rates (IR) and recovery rates (RR) for each time step in the treatment group and the control group

Strain group	Time-step	Non-teated group				IR	RR	Treatment group					
		# at risk	# to recover	# new cases	# reco-vered			# at risk	# to recover	# new cases	# reco-vered	IR	RR
RIF	0–2	5	3	0	2	0.00	0.50	6	2	5	0	0.71	0.00
	2–4	7	1	0	1	0.00	1.00	1	7	0	4	0.00	0.40
	4–6	8	0	0	0	0.00		5	3	1	1	0.11	0.20
	6–8	8	0	0	0	0.00		5	3	0	3	0.00	1.00
	8–10	8	0	0	0	0.00		8	0	0	0	0.00	
RIF-GEN-SUL	0–2	6	2	0	1	0.00	0.33	6	2	3	0	0.33	0.00
	2–4	7	1	0	1	0.00	1.00	3	5	1	2	0.20	0.25
	4–6	8	0	0	0	0.00		4	4	0	2	0.00	0.33
	6–8	8	0	2	0	0.14		6	2	0	2	0.00	1.00
	8–10	6	2	0	2	0.00	1.00	8	0	0	0	0.00	

## Discussion

There is overwhelming evidence that the continuous use of antibiotics in food animals increases the number of resistant bacteria in their intestine [5, 6, 8, 18–23]. However, it remains to be shown whether this is caused by selection of resistant bacteria, already present in treated animals only, or whether treatment also promotes colonization of more animals with resistant bacteria. This has not been previously analyzed in pigs, and it represented the main goal of the present work.

In a previous study the strain *E*. *coli* 912, of pig origin, was orally administered to human volunteers and results showed that, even though the sulphonamide resistance encoded by the isolate was not found to be transferred to the commensal microbiota, the strain was able to colonize the human gut. It was also proven that once in the intestine, the bacteria could survive for a long period, enabling the possible transfer of resistance genes to the commensal bacteria in the gastrointestinal human tract [11]. In this study we analyzed the potential spread of the same strain during an in vivo experiment in pigs treated and non-treated with apramycin in order to elucidate how the selection force of antibiotic treatment affects spread of resistant bacteria. The plasmid-located gene responsible for apramycin resistance in this strain was *aac*(*3*)-*IV*. The experiment was performed only once, which represents a study limitation, and therefore the statistical assessment carried out can be only descriptive in nature. The incidence rates appeared lower in the non-treated groups compared to the treated groups (Table 2), however, no statistical testing could be performed to confirm the trend. A larger-scale study including more animals per group would be required to prove whether the incidence rates are indeed different. Recovery did not appear to be different, which is an effect of the removal of the antimicrobial.

Results from our study revealed selection from treatment with apramycin in the intestinal microbiota of treated pigs, leading to significantly higher counts of resistant strains than in pigs that did not receive the antibiotic. On average, selection resulted in differences in CFU of *E*. *coli* 912 in the feces of apramycin-treated and non-treated pigs of around 100-fold. Unlike reported by Trobos et al. [11] for human volunteers, we found that the strain was relatively poor in colonizing the gut of the pigs, in the sense that the peak in CFU was reached already on day 3 of treatment (day 2) in both groups. Several factors might have contributed to the rapid loss of *E*. *coli* 912. Even though the rifampicin resistance mutation does not affect the fitness of the mutant strain “in vitro”, there could be a fitness cost “in vivo” due to this mutation as previously described [37]. Further experiments where both wild type and RIF-R strains are administered to pigs should be performed in order to elucidate this premise. It has previously been observed that predominant *E*. *coli* clones normally associated with the individual intestinal microbiota make it difficult for introduced strains to establish themselves permanently [38–42]. Interestingly, in a previous study performed in calves, natural conjugative apramycin resistance plasmid isolated from commensal organisms of newborn calves was found to confer a fitness advantage on new hosts cells [43]. However, the methodology in the current study did not allow for estimation of plasmid transfer to other bacteria. Cavaco et al. [34] also demonstrated that the administration of several β-lactams (ceftiofur, cefquinome and amoxicillin) led to significantly higher counts of antimicrobial resistant strains compared to the control group. However, contrary to our results, the study which was set up very similar to ours, showed that the selective effects exerted by these antibiotics persisted for longer periods and cefotaxime-nalidixic resistant strains were detected up to 15–20 days after inoculation of the strain in all the groups, no matter whether the antibiotic was administered or not.

In a previous study it was concluded that apramycin administration is most probably driving the increasing occurrence of apramycin/gentamicin cross-resistance in swine [44]. Moreover, this increasing occurrence in animals is of concern and should be under close surveillance. Resistance to apramycin and gentamicin in *Enterobacteriaceae* and other enteric pathogens usually remains low in pigs at slaughter and in food at retail [44]. Notably, several studies from Great Britain have shown that ca. 26% of the gentamicin-resistant pathogenic *E*. *coli* strains from humans were carrying the *aac*(*3*)-*IV* gene [45, 46].

Strain *E*. *coli* 912 has now been well characterized with respect to colonization of both pigs and humans, which might make it a suitable challenge strain in studies on aspects of *E*. *coli* microbiota of pigs. To deeply study the main features of this strain whole genome sequencing was performed. Apart from the genes *aac*(*3*)-*IV* and *sul2*, sequencing revealed that the strain also harbored the genes *bla*_TEM-1_, *strA/B*, *aph*(*4*) and *tet*(*X*). Southern blot hybridization analysis showed that all the resistance genes but *tet*(*X*) were carried in the same plasmid that was proven to be conjugative (not shown). Thus, treatment with any of the antibiotics for which the strain is resistant may co-select for the selection and spread of all the resistance genes as a whole since they can be transmitted within the same transferable element. With this knowledge, this strain also constitutes a suitable candidate for studies of how resistance plasmids contribute to the distribution of resistance genes in the intestine, as well as for the analysis of rates and mechanisms of transfer of such plasmids.

Whole genome sequencing also revealed that *E*. *coli* 912 harbors virulence genes, encoding functions related to microcin synthesis (*mchB/C/F* and *mcmA*), toxin production (*cnf1*), fimbriae synthesis (*lpfA*, *prfb*), iron uptake (*iroN*), increased serum survival (*iss*) and hemoglobin-binding protease (*tsh*) (Table 2). Since virulence genes responsible for pathogenicity are often located on transmissible genetic elements [47], *E*. *coli* 912 may represent a source of such virulence determinants, which could disseminate to pathogenic subgroups of *E*. *coli*. However, it is important to stress that the strain has been given to both humans and pigs without sign of symptoms, indicating that it is a well-characterized strain which can be safely used in future studies.

Other studies have reported that antibiotic treatment influences selection, spread and persistence of resistant bacterial members of the family *Enterobacteriaceae* [48, 49]. A prospective in vivo/in situ study demonstrated that the administration of low-dose in-feed oxytetracycline of chickens and farm dwellers did not only lead to colonization of the intestinal microbiota of chickens with tetracycline-resistant *E*. *coli* strains but also acquisition of such resistance in *E*. *coli* in the gut of the farm family [50].

The quantitative data generated by this study might be useful for assessment of the risk of acquisition of antimicrobial resistance from aminoglycosides use in pig production. Besides, this is the first study providing evidence that the selection of resistant bacteria by treatment translates into spread between pigs and that antibiotic administration enhances the risk of transfer among treated animals. Further large-scale studies including more pigs per group, analysis of the immune status of the pigs, and analysis of the *E*. *coli* resistance gene pool in the gut of the pigs at the start of the experiments as well as analysis of the variation of both aspects between piglets and between pens should be performed to confirm our conclusions. All these factors might have an impact on strain colonization, shedding and spread of the strain, as well as on emergence and spread of antimicrobial resistance. As mentioned, gentamicin is used for treatment of critical human systemic infections, such as bacteremia. Due to the risk of transfer of gentamicin resistance genes or gentamicin-resistant *E*. *coli* from animals to humans and the consequent risk of difficulty in treating infections with gentamicin-resistant *E*. *coli*, the selective force conferred by apramycin for presence of gentamicin-resistant *E*. *coli* in animals and the potential enhanced spread among them is of great concern.

## Additional files

10.1186/s13567-015-0291-z **Growth of the** ***E. coli*** **912 strain and the derivative RIF-R mutant.** The growth of the strains was followed over a period of 24 h. A similar growth was observed.
10.1186/s13567-015-0291-z **Xba** **I macrorrestriction-PFGE analysis.** *Xba*I-profiles of E. coli 912 (1) and representative *E. coli* 912-like strains (2–11). M: λ ladder PFGE marker (New England Biolabs) was used as molecular size standard.
10.1186/s13567-015-0291-z **Following of the spread of the strain E. coli 912.** Selection with rifampicin (A) and rifampicin- gentamicin-sulphonamide (B), respectively. *Pigs inoculated in both groups.
