# Supplementary figures and images for: Apramycin treatment affects selection and spread of a multidrug-resistant Escherichia coli strain able to colonize the human gut in the intestinal microbiota of pigs
Source: Vet Res. 2016 Jan 7;47:12. doi: 10.1186/s13567-015-0291-z (PMC4704421; doi:10.1186/s13567-015-0291-z)

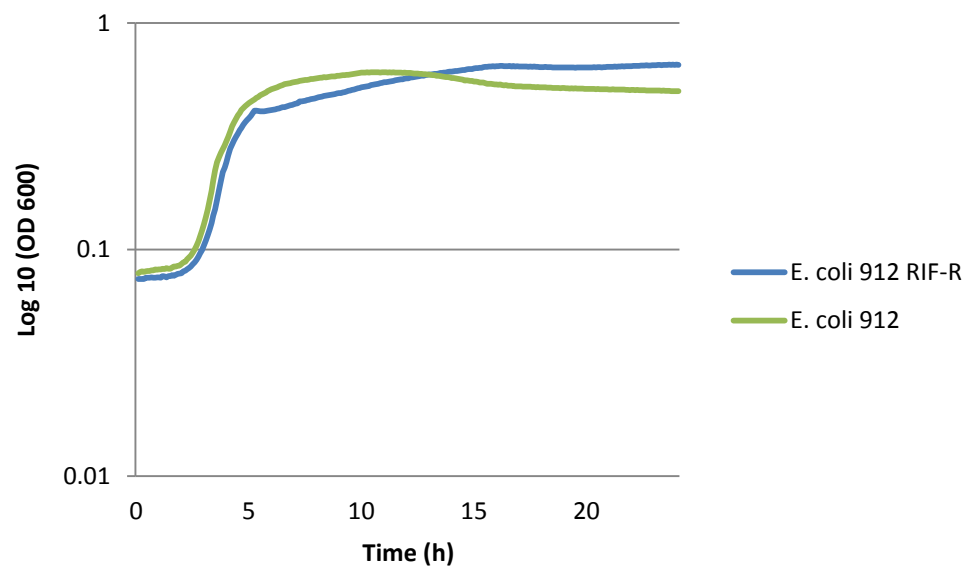

Supplement: Supplementary file 1 — 10.1186/s13567-015-0291-z Growth of the E. coli 912 strain and the derivative RIF-R mutant. The growth of the strains was followed over a period of 24 h. A similar growth was observed. [file 13567_2015_291_MOESM1_ESM.pdf]

M 1 2 3 4 5 6 7 8 9 10 11

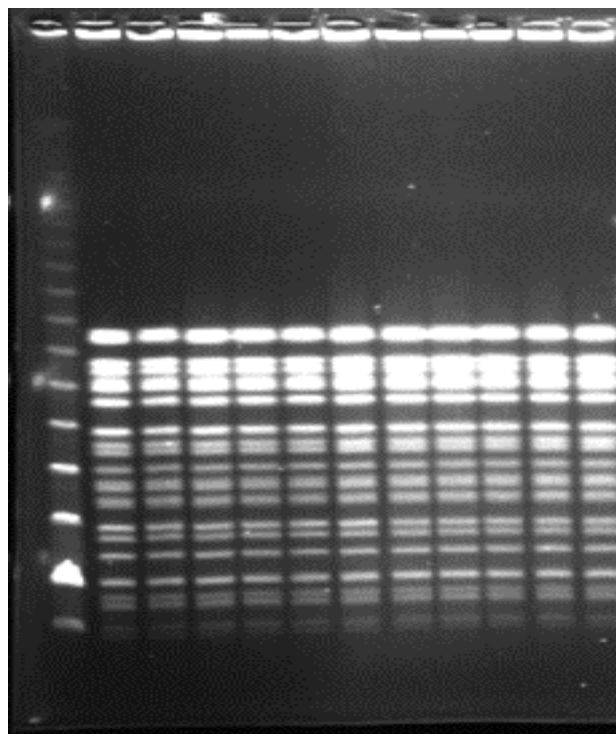

Supplement: Supplementary file 2 — 10.1186/s13567-015-0291-z Xba I macrorrestriction-PFGE analysis. XbaI-profiles of E. coli 912 (1) and representative E. coli 912-like strains (2–11). M: λ ladder PFGE marker (New England Biolabs) was used as molecular size standard. [file 13567_2015_291_MOESM2_ESM.pdf]
