# Supplementary material for: Apramycin treatment affects selection and spread of a multidrug-resistant Escherichia coli strain able to colonize the human gut in the intestinal microbiota of pigs
Source: Vet Res. 2016 Jan 7;47:12. doi: 10.1186/s13567-015-0291-z (PMC4704421; doi:10.1186/s13567-015-0291-z)
Supplement: Supplementary file 3 — 10.1186/s13567-015-0291-z Following of the spread of the strain E. coli 912. Selection with rifampicin (A) and rifampicin- gentamicin-sulphonamide (B), respectively. *Pigs inoculated in both groups. [file 13567_2015_291_MOESM3_ESM.pdf]

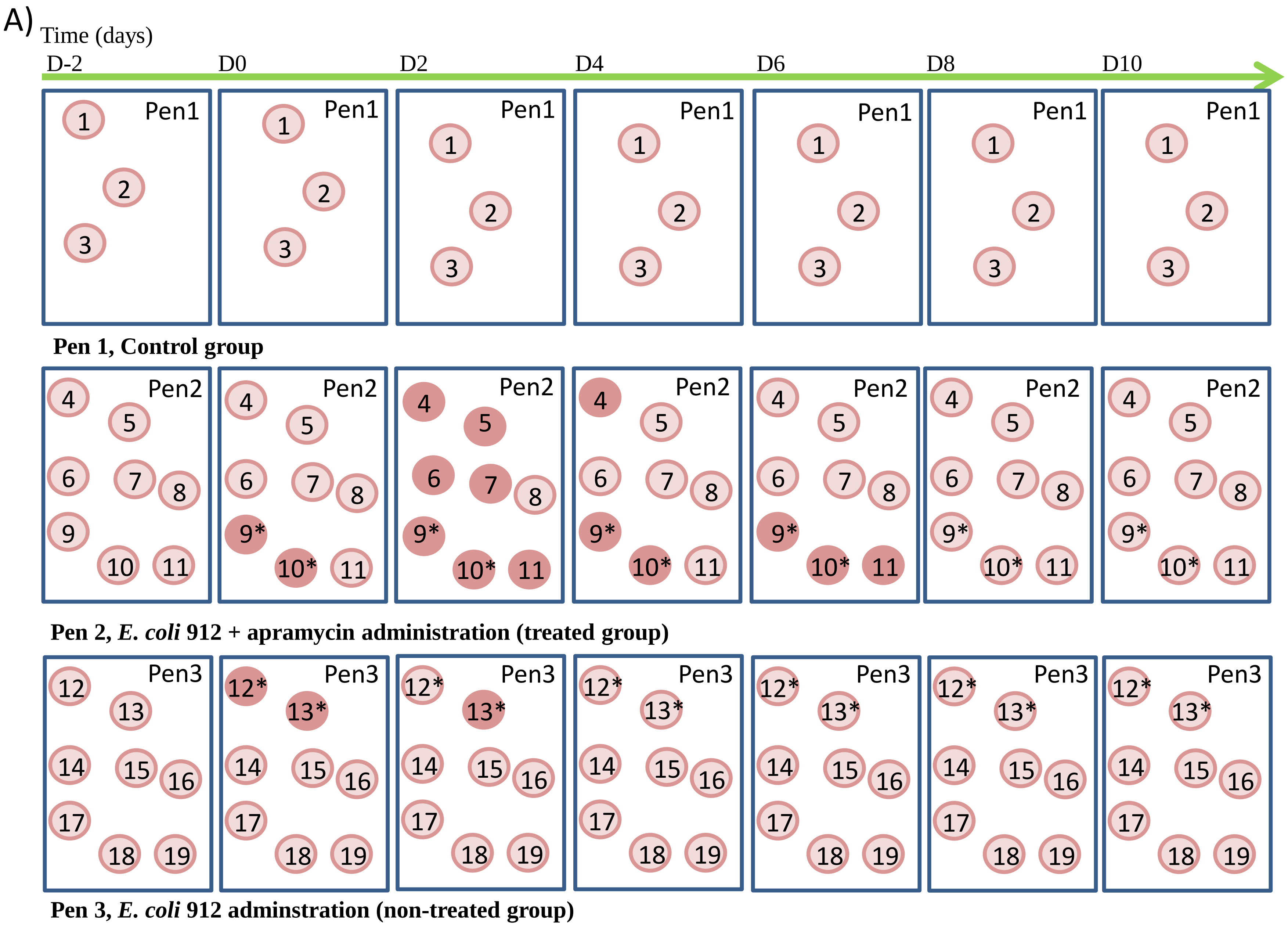
A)
Time (days)
D-2
D0
D2
D4
D6
D8
D10
Pen1
1
2
3
Pen 1, Control group
Pen2
4
5
6
7
8
9
9*
10
10*
11
Pen 2, E. coli 912 + apramycin administration (treated group)
Pen3
12
12*
13
13*
14
15
16
17
18
19
Pen 3, E. coli 912 adminstration (non-treated group)

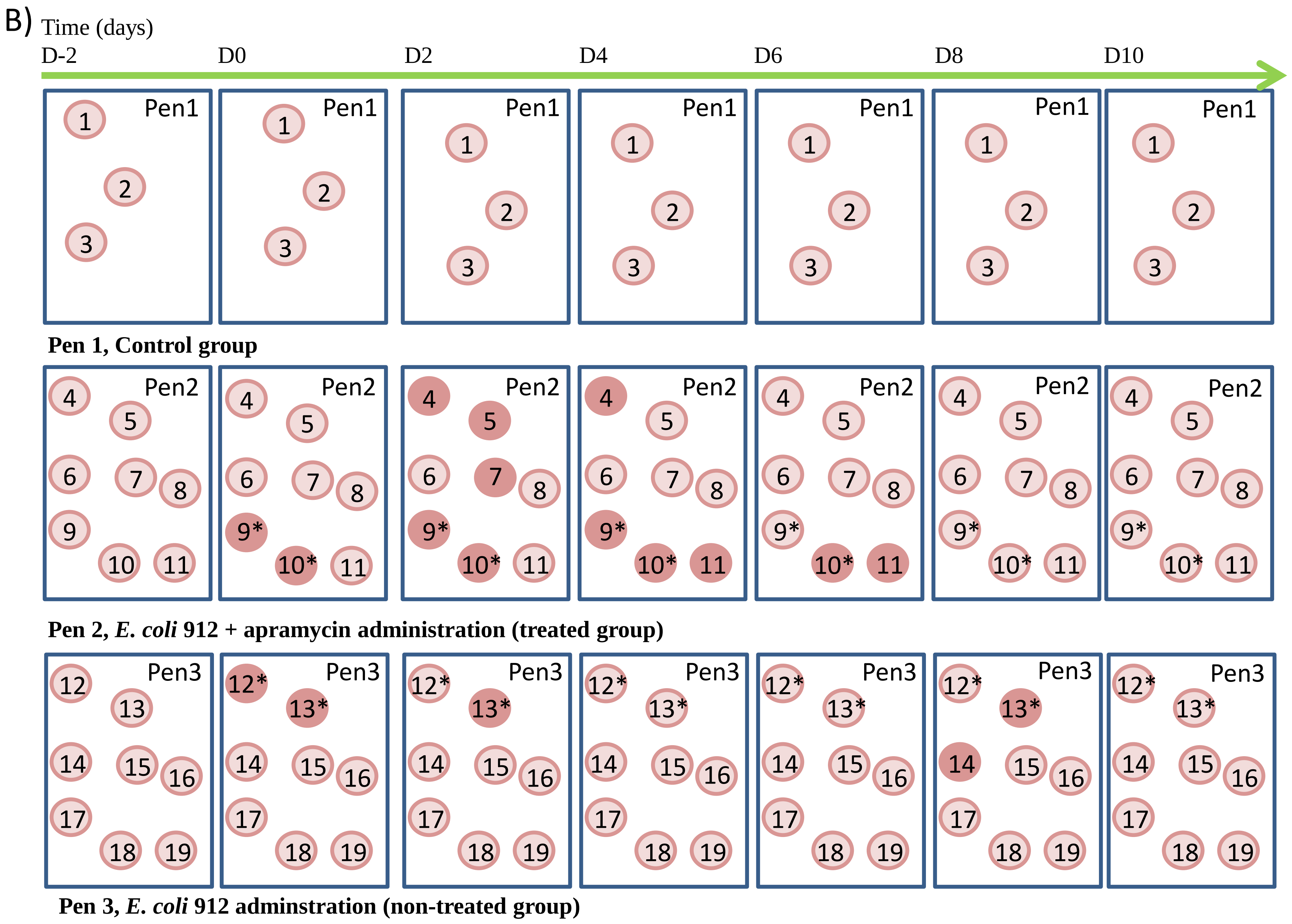
B)
Time (days)
D-2
D0
D2
D4
D6
D8
D10
Pen 1, Control group
Pen 2, *E. coli* 912 + apramycin administration (treated group)
Pen 3, *E. coli* 912 adminstration (non-treated group)
